# IL-6-Mediated Upregulated miRNAs in Extracellular Vesicles Derived from Lund Human Mesencephalic (LUHMES) Cells: Effects on Astrocytes and Microglia

**DOI:** 10.3390/biom13050718

**Published:** 2023-04-22

**Authors:** Kento Nishi, Hiroto Izumi, Taisuke Tomonaga, Chikage Nagano, Yasuo Morimoto, Seichi Horie

**Affiliations:** 1 Center for Stress-Related Disease Control and Prevention, University of Occupational and Environmental Health, Kitakyushu 807-8555, Japan; 2 Department of Health Policy and Management, Institute of Industrial Ecological Sciences, University of Occupational and Environmental Health, Kitakyushu 807-8555, Japan; 3 Department of Occupational Pneumology, Institute of Industrial Ecological Sciences, University of Occupational and Environmental Health, Kitakyushu 807-8555, Japan

**Keywords:** IL-6, microRNA, extracellular vesicles, neural precursor cells, astrocyte, microglia, stress, depression

## Abstract

Psychological stress plays a major role in depression, and interleukin-6 (IL-6) is elevated during depression and psychological stress. MicroRNAs (miRNAs) in extracellular vesicles (EVs), including exosomes and microvesicles, suppress mRNA expression in other cells when endocytosed. In this study, we analyzed the effect of IL-6 on EVs secreted by neural precursor cells. Cells from the human immortalized neural precursor cell line LUHMES were treated with IL-6. EVs were collected using a nanofiltration method. We then analyzed the uptake of LUHMES-derived EVs by astrocytes (ACs) and microglia (MG). Microarray analysis of miRNAs was performed using EV-incorporated RNA and intracellular RNA from ACs and MG to search for increased numbers of miRNAs. We applied the miRNAs to ACs and MG, and examined the cells for suppressed mRNAs. IL-6 increased several miRNAs in the EVs. Three of these miRNAs were originally low in ACs and MG (hsa-miR-135a-3p, hsa-miR-6790-3p, and hsa-miR-11399). In ACs and MG, hsa-miR-6790-3p and hsa-miR-11399 suppressed four mRNAs involved in nerve regeneration (NREP, KCTD12, LLPH, and CTNND1). IL-6 altered the types of miRNAs in EVs derived from neural precursor cells, by which mRNAs involved in nerve regeneration were decreased in ACs and MG. These findings provide new insights into the involvement of IL-6 in stress and depression.

## 1. Introduction

The global prevalence of depression and depressive symptoms has increased in recent decades, with the prevalence of the disorder ranging from 20% to 25% in women and 7% to 12% in men [1,2]. The main diagnostic criteria for depression are based on the Diagnostic and Statistical Manual of Mental Disorders, Fifth Edition, but these criteria are episodic and tend to be subjective in their assessment [3]. In recent years, attention has been focused on the theory that stress induces an inflammatory response that leads to changes in the neural structure as part of the pathophysiology of depression [4]. The number of glial cells in the prefrontal cortex is markedly reduced in depressed patients [5]. Among glial cells, astrocytes (ACs) form contacts with many synapses and regulate neuronal activity through the release of glial transmitters such as adenosine triphosphate [6]. In postmortem tissues of depressed patients, gene expression is markedly decreased in ACs [7], and proinflammatory cytokines elicited by microglia (MG) have been implicated in the development of depression [8].

IL-6 is a type of proinflammatory cytokine that is prominently elevated during infectious diseases, trauma, and autoimmune diseases and is a marker of the inflammatory response [9]. IL-6 is produced by brown adipose tissue in response to sympathetic nerve stimulation [10], which is independent of adrenal corticosteroids and is therefore also attracting attention as a stress biomarker [8,11]. It is also elevated during psychiatric disorders such as depression [12,13].

Extracellular vesicles (EVs) are secreted (exosomes, microvesicles) or formed (apoptotic vesicles) from various cells and different EV types can be distinguished by their size, content, and mechanism of production [14]. EVs contain proteins and nucleic acids, such as microRNA (miRNA) and mRNA, and enter cells predominantly via clathrin-independent endocytosis and macropinocytosis [15,16,17]. This suggests that EVs play a role in the transmission of information to distant cells. miRNAs are noncoding RNAs of 20–25 nucleotides in length that bind to mRNAs and suppress gene expression by degrading mRNAs or inhibiting their translation [14]. Many studies have focused on the miRNAs present in EVs and analyzed their qualitative and quantitative changes [18,19]. For example, astrocytes stimulated by IL-1β or TNF-α were reported to release EVs containing miRNAs (miR-125a-5p and miR-16-5p) that are involved in neuronal survival, differentiation, and neurite outgrowth [20]. This suggests that the miRNAs in EVs are involved in neuronal function. As mentioned above, IL-6 is elevated during chronic stress and may affect these neurons. However, it remains unclear how IL-6 affects EV secretion and the qualitative and quantitative responses of EVs to it. In this study, we used a nanofiltration method to isolate EVs secreted from cultured neural precursor cells and analyzed the quantitative and qualitative responses of miRNAs to IL-6.

## 2. Materials and Methods

### 2.1. Cell Culture

An immortalized human dopaminergic neuronal precursor cell line (LUHMES cells), immortalized human microglia (IHMG), and immortalized human astrocytes (IHACs) were purchased from Applied Biological Materials Inc. (Richmond, BC, Canada). LUHMES cells, IHACs, and IHMG were maintained in DMEM/F12 medium, Prigrow IV medium, and Prigrow III medium, respectively. All cells were cultured in medium with 10% heat-inactivated fetal bovine serum (FBS) and 1% (*v*/*w*) penicillin/streptomycin, and were maintained at 37 °C with 5% CO_2_. IL-6 (HZ-1019) was purchased from Proteintech Group, Inc. (Rosemont, IL, USA). Cells were treated with IL-6 at 0.1 or 1 μg/mL and collected 24 h later.

### 2.2. Preparation of Nluc-Fused CD81 Plasmids and Their Expression

The construction of nanoluciferase (Nluc)-fused CD81 plasmids that co-express GFP and puromycin was performed as described previously [21]. Nonproliferative lentivirus was packaged using the System Biosciences lentivirus packaging system. Briefly, nanoluciferase (Nluc)-fused CD81 plasmids were mixed with pPACKH1 plasmid mix (pPACKH1-GAG, pPACKh1-REV, pVSV-G) and transfected into COS1 cells using X-tremeGENE™ 9 DNA Transfection Reagent (Roche Molecular Biochemicals, Penzberg, Germany). After 36 h, the culture medium was filtered through a 0.45 µm syringe filter and added directly to the LUHMES cells. Infected cells were cultured with 10 µg/mL puromycin (ant-pr; InvivoGen, San Diego, CA, USA) for more than 2 weeks to establish CD81-Nluc-expressing LUHMES cells.

### 2.3. Fractionation and Isolation of EVs in Cell Culture Medium Using Nanofiltration

**Figure 1:**
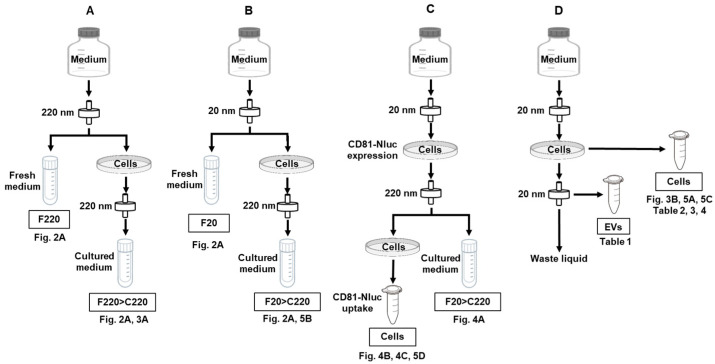
Preparation of media used in this experiment and samples used in the assay. This research mainly consisted of four methods (**A**–**D**). (**A**,**B**) show the sample preparation for ELISA analysis, (**C**) that for Nluc activity analysis, and (**D**) that for RNA analysis. F220 and F20 indicate fresh medium passed through 220 and 20 nm filters, respectively. C220 is medium filtered at 220 nm after culture. F220 > C220 and F20 > C220 refer to samples obtained by culturing cells in F220 or F20 medium, respectively, and subsequently filtering the cell medium using a 220 nm syringe filter. Adjusted samples were used in the experiments as indicated by the numbers in each figure.

Isolation of EVs using a syringe filter was performed as described previously [17]. Syringe nanofilters of 220 nm (SFPES013022N; Membrane Solutions), 100 nm (SFPES013010N; Membrane Solutions), 50 nm (SF16008; TISCH Scientific), and 20 nm (ANPEL Laboratory Technologies, Shanghai, China) were used in this study. Figure 1 shows a schematic of sample preparation. F and C in front of the numbers indicate fresh medium and culture supernatant, respectively. FBS in all cell culture media was filtered at 220 nm (F220) or 20 nm (F20) to remove visible solids and apoptotic bodies derived from the FBS. In some experiments, F220 medium was used, while in others F20 medium was used. The method of sample preparation is described in detail for each analysis. As a common technique, when passing the medium through a syringe filter of 100 nm or less, a rate of one drop per second was strictly employed.

### 2.4. ELISA Analysis with Anti-CD9, Anti-CD63, and Anti-CD81 Antibodies

The indicated numbers of LUHMES cells, ACs, and MG were seeded in a plate dish. The next day, 1 or 10 pg/mL IL-6 was added and the medium was collected 24 h later. The collected medium was passed through a 220 nm filter and then analyzed immediately. Tetraspanins in both fresh medium and culture supernatant were quantified using an ELISA kit (296-83701 for CD9, 290-83601 for CD63, and 292-83801 for CD81; Wako, Osaka, Japan). A sample volume of 50 or 100 μL was used, depending on the amount of tetraspanins in the sample and the sensitivity of the antibody. Reporter lysis buffer was added to the cells in the dish and each sample was transferred to a tube. This tube was vortexed for 20 s and centrifuged at 10,000× *g* for 2 min. An appropriate amount of supernatant was transferred to a 96-well white plate and mixed with an equal volume of CellTiter-Glo 2.0 Cell Viability Assay (Promega, Madison, WI, USA) reagent. After 5 min, ATP activity was measured using a luminometer (Luminescencer JNII RAB-2300; ATTO, Tokyo, Japan). The values obtained via ELISA were normalized using the values of cellular ATP activity as an internal standard.

### 2.5. Western Blot Analysis with Immunoprecipitation

Each cell line was sonicated in lysis buffer consisting of 50 mM Tris-HCl (pH 8.0), 1 mM EDTA, 120 mM NaCl, 0.5% Nonidet P-40, and 0.5 mM PMSF, and centrifuged at 21,000× *g* for 10 min at 4 °C. Proteins (1 mg) were immunoprecipitated with 5 μg of anti-IL-6R antibody (ab222101, Lot GR34013 9-1; Abcam, Cambridge, UK) or rabbit IgG for 4 h at 4 °C. Immune complexes were isolated by incubation with 10 μL of Protein A/G PLUS-Agarose (sc-2003; Santa Cruz Biotechnology, Santa Cruz, CA, USA) for 1 h at 4 °C. Protein A/G-agarose pellets were washed twice with 1 mL of lysis buffer. Each sample was subjected to SDS-PAGE and then transferred to a PVDF membrane. IL-6R was detected using anti-IL-6R antibody (ab222101, 1/1000 dilution) in PBS-T (Takara, Tokyo, Japan) containing 1% BSA Fraction V (Rockland Immunochemicals, Limerick, PA, USA) overnight at 4 °C. Bound secondary anti-rabbit IgG, HRP-Linked Whole Ab Donkey (NA934; Cytiva Life Sciences, Marlborough, MA, USA), was visualized using an enhanced chemiluminescence kit (GE Healthcare Bio-Sciences, Marlborough, MA, USA).

### 2.6. Uptake of EVs and Nluc Activity Assay

Samples for Nluc activity measurement were prepared using two methods.

(1) LUHMES cells expressing CD81 were digested with trypsin. After neutralization with medium, cells were washed twice with PBS and then resuspended in PBS. Suspended cells were passed through 220, 100, 50, and 20 nm syringe filters, and Nluc activity after each filtration was measured. The cell suspension before passing through a filter and the supernatant obtained by centrifugation (2000× *g* for 2 min) were also analyzed. The measurements were performed by mixing 100 μL of each sample with 100 μL of Nano-Glo Luciferase Assay System (Promega) reagent. Nluc activity was measured using a luminometer. (2) The culture supernatant of LUHMES cells expressing CD81-Nluc was passed through a 220 nm syringe filter, and IHACs and IHMG were cultured in this medium for the indicated times. Cells were washed twice with PBS and lysed in reporter lysis buffer. After collection, the lysate was vortexed (20 s) and then centrifuged (12,000 rpm for 2 min). The supernatant (100 μL) was mixed with Nano-Glo Luciferase Assay System reagent and light intensity was measured using a luminometer. To prepare a background sample, after adding the culture supernatant containing CD81, cells were immediately washed and lysed as described above. The supernatant (20 μL) was also mixed with CellTiter-Glo 2.0 Cell Viability Assay reagent and light intensity was measured with a luminometer. The value of Nluc activity was normalized using the level of cellular ATP activity as an internal standard.

### 2.7. Reverse Transcription and Quantitative Real-Time PCR Analysis (qRT-PCR)

The indicated numbers of LUHMES cells, ACs, and MG were seeded in a plate dish. The next day, 1 or 10 pg/mL IL-6 was added and cells were collected 24 h later. Total RNA including miRNA was purified with an miRNeasy Kit (217084; Qiagen, Venlo, the Netherlands). qRT-PCR assay was then performed as described previously [22], for which the primer sets were as follows: Hs00233521_m1 for CD9, Hs01041238_g1 for CD63, Hs01002167_m1 for CD81, Hs01075664_m1 for IL-6R, and Hs01060665_g1 for β-actin (Applied Biosystems, Foster City, CA, USA). Values were normalized to those of human β-actin. The comparative cycle time method was used to quantify gene expression. All samples were analyzed in duplicate in each experiment.

### 2.8. miRNA Extraction

The indicated numbers of LUHMES cells, ACs, and MG were seeded in a plate dish. The next day, 1 or 10 pg/mL IL-6 was added and the medium was collected 24 h later. miRNA extraction was performed as described previously [21]. Briefly, total RNA of EVs captured with a 20 nm filter was eluted with ISOGEN (Nippon Gene, Tokyo, Japan) and purified using a NucleoSpin miRNA Plasma Kit (Macherey-Nagel, Düren, Germany).

### 2.9. miRNA Transfection

ACs and MG were transfected with the following miRNAs: hsa-miR-135-3p, hsa-miR-6790-3p, hsa-miR-11399, and control miRNA (Invitrogen, Carlsbad, CA, USA). Briefly, each 25 pmol miRNA was mixed with 7.5 μL Lipofectamine RNAiMAX (Invitrogen) in 100 μL Opti-MEM. The miRNA complexes were applied to 2 × 10^5^ cells. After 24 h, cells were collected and miRNA microarray analysis was performed.

### 2.10. Microarray Analysis

Microarray analysis of miRNAs has been described previously [17]. Briefly, a Human miRNA Oligo chip-4 plex (TORAY, Tokyo, Japan) containing 2565 probe sets was used. Microarray analysis of mRNAs has been described previously [23]. Briefly, a Human Oligo chip 25k set (TORAY) containing 24,460 probe sets was used. Each microRNA and mRNA was normalized using the global normalization method, which adjusted the median of the detected signal intensity to 25.

### 2.11. Statistical Analysis

Statistical analysis was performed using JMP pro 15 (SAS Institute Inc., Cary, NC, USA). The results were compared using Tukey’s range test. Data are expressed as the mean ± SD. *p* < 0.05 was considered to indicate a statistically significant difference.

## 3. Results

### 3.1. Purification of EVs Using Nanofiltration

In our previous study, EVs fractionated using nanofiltration using a 50 nm syringe filter were analyzed, with the following results being obtained [21]. The EVs were spherical, as determined using transmission electron microscopy (TEM), with a diameter of approximately 170 nm, as determined using dynamic light scattering (DLS). Furthermore, the EVs contained CD9, CD63, CD81, and EpCAM proteins, as determined using Western blotting, and contained miRNAs of less than 30 bp in length, as revealed by an Agilent 2100 Bioanalyzer. EVs captured with a 50 nm syringe filter could be collected by reverse flow, but EVs captured with a 20 nm syringe filter could not. Therefore, we are unable to show the analytical results for EVs captured with a 20 nm syringe filter, but expect similar results to those captured with a 50 nm syringe filter.

**Figure 2:**
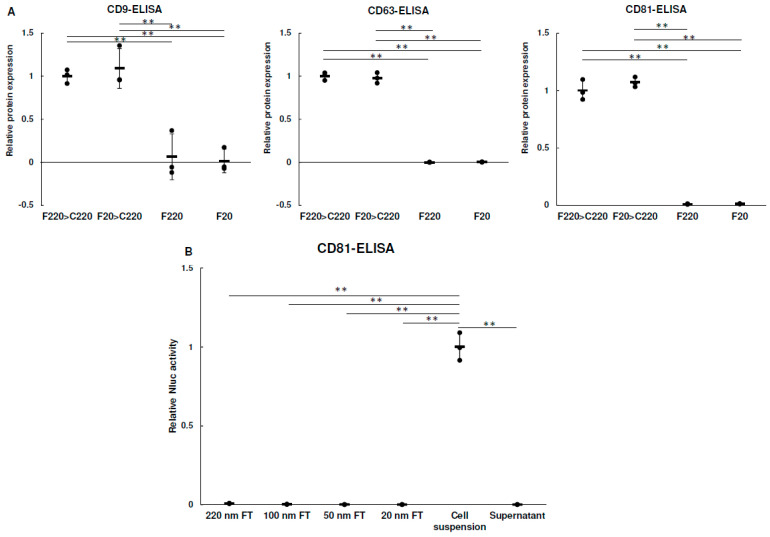
Evaluation of EVs using nanofiltration. (**A**) A total of 4 × 10^5^ cells were seeded in a six−well plate. The next day, cells were washed with PBS and supplemented with 1 mL of F220 or F20. After 24 h, culture medium was collected and passed through a 220 nm filter. Each tetraspanin in the medium was measured using ELISA and each value was normalized using the cellular ATP activity as an internal standard. Data were obtained from three independent experiments (*n* = 3), ** *p* < 0.01. Error bars represent standard deviation. (**B**) A total of 1 × 10^4^ LUHMES cells expressing CD81−Nluc were prepared. Luciferase activity in flow−through (FT) was measured after filtration of CD81−Nluc. The filter pores were 20, 50, 100 and 220 nm in diameter. Cell suspensions were measured with intact cells prior to filtration. Cell suspensions were centrifuged at 800× *g* for 5 min and supernatant was also measured. Data were obtained from three independent experiments (*n* = 3), ** *p* < 0.01. Error bars represent standard deviation.

In accordance with the method described in Figure 1, F220 > C220, F20 > C220, F220, and F20 samples were prepared, and CD9, CD63, and CD81 protein levels in each sample were measured (Figure 2A). CD9, CD63, and CD81 were all found in the F220 > C220 and F20 > C220 samples, whereas almost no expression was observed in the F220 and F20 samples. These results suggest that the antibody used in this study recognized human tetraspanins, but not bovine tetraspanins, or that there were very few EVs derived from bovine serum.

Next, we examined whether nanofiltration damaged EVs. CD81-Nluc-expressing cells were suspended in PBS and Nluc activities in PBS passed through 220, 100, 50, and 20 nm syringe filters were measured. Since Nluc is released outside the cells when the cells are damaged during passage through the syringe filter, cell damage can be confirmed by measuring the Nluc activity of the sample after passage. As shown in Figure 2B, no Nluc activity was observed in any of the PBS samples, even when the smallest filter (20 nm) was used. These results suggest that the cells suffered little damage when passaged at a rate of less than two drops per second, as described in the Methods section. Therefore, it was considered that EVs, which are much smaller than cells, were hardly damaged by pressurization when passing through the filter.

### 3.2. The Expression of Tetraspanin Proteins and mRNAs

**Figure 3:**
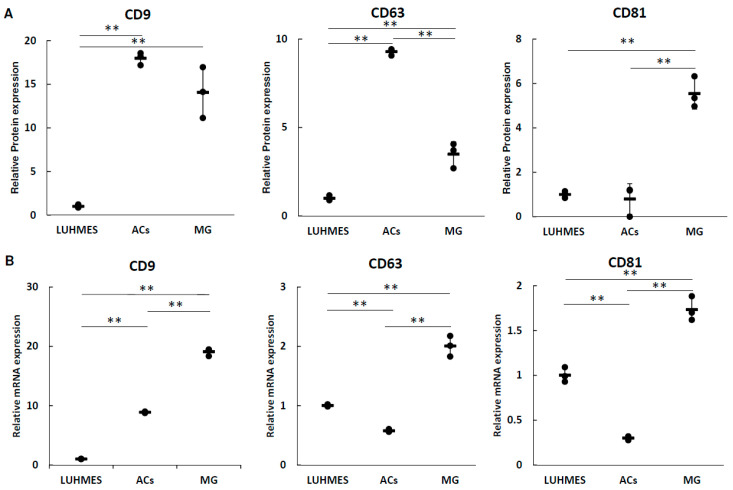
Expression analysis of tetraspanins associated with EVs. (**A**) A total of 2 × 10^5^ cells were seeded in a 12-well plate. The next day, the cells were washed with PBS and supplemented with 1 mL of F220. After 24 h, culture medium was collected and passed through a 220 nm filter. Each tetraspanin in the medium was measured using ELISA and each value was normalized using the cellular ATP activity as an internal standard. Data were obtained from three independent experiments (*n* = 3), ** *p* < 0.01. Error bars represent standard deviation. (**B**) A total of 1 × 10^6^ cells were seeded into a 60 mm dish. The next day, the cells were washed with PBS and supplemented with 3 mL of F20. After 24 h, the cells were collected and total RNA was purified. The expression levels of each tetraspanin in each cell type were analyzed using qRT-PCR. Data were obtained from three independent experiments (*n* = 3), ** *p* < 0.01. Error bars represent standard deviation.

The expression of tetraspanin proteins in media of LUHMES cells, ACs, and MG was measured using ELISA (Figure 3A), and the expression of each tetraspanin mRNA in cells was analyzed using real-time PCR (Figure 3B). There was no correlation between tetraspanin protein expression in the medium and cellular mRNA expression in each cell. These analyses confirmed that all cells expressed three tetraspanins.

### 3.3. Uptake of LUHMES-Derived EVs by Astrocytes (ACs) and Microglia (MG)

**Figure 4:**
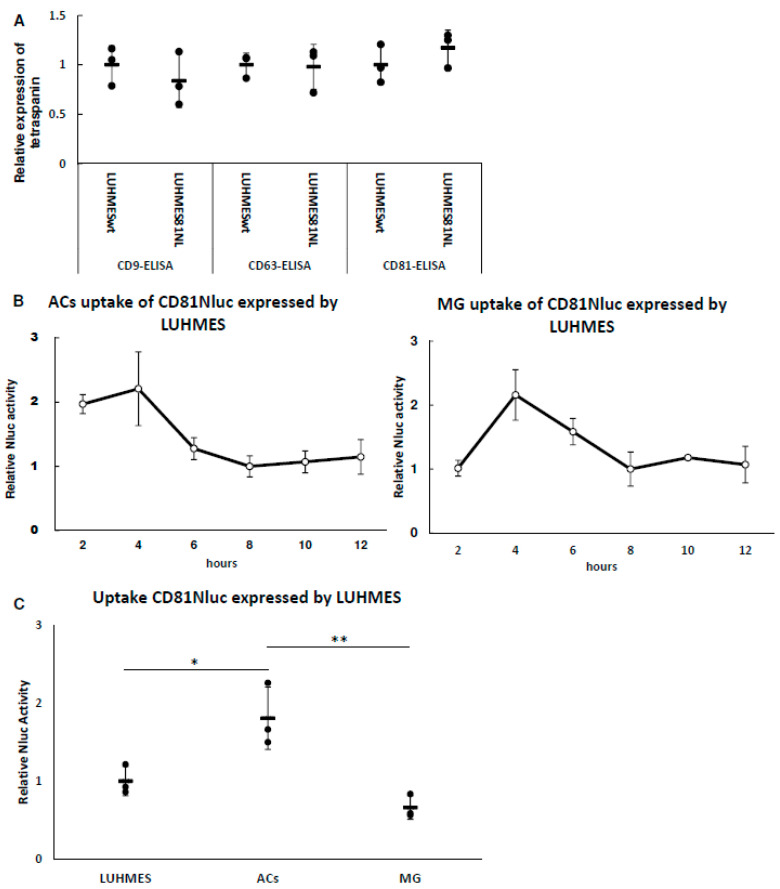
Uptake of LUHMES-derived EVs by ACs and MG. (**A**) A total of 2 × 10^5^ cells were seeded in a 12-well plate. The next day, the cells were washed with PBS and supplemented with 1 mL of F20. After 24 h, culture medium was collected and passed through a 220 nm filter. Each tetraspanin in the media of CD81-Nluc transgenic LUHMES and wild-type LUHMES cells was measured using ELISA. These values were normalized using the cellular ATP activity of each cell. Data were obtained from three independent experiments (*n* = 3). Error bars represent standard deviation. (**B**) A total of 2 × 10^5^ cells were seeded into a 12-well plate and cultured with medium containing LUHMES-derived CD81-Nluc expressed in EVs for the indicated time. The background was subtracted from each value of Nluc activity and the result was normalized using cellular ATP activity as an internal standard. The value after 8 h was set to 1 for each cell. Data were obtained from three independent experiments (*n* = 3). Error bars represent standard deviation. (**C**) Uptake of LUHMES-derived CD81-Nluc expressed in EVs in each cell was quantified 8 h after administration from the results of (**B**). Data were obtained from three independent experiments (*n* = 3), * *p* < 0.05, ** *p* < 0.01. Error bars represent standard deviation.

EVs derived from cells are taken up by other cells and their contents, including proteins and RNAs, are transferred into the cells and function there. Culture medium of LUHMES (neural progenitor) cells expressing CD81-Nluc was added to ACs and MG, and the uptake of EVs containing CD81-Nluc in each cell was examined. Before uptake analysis, we examined whether the expression of each endogenous tetraspanin was altered by the expression of CD81-Nluc. As shown in Figure 4A, the expression of each tetraspanin in the EVs was not significantly different after expression of the CD81-Nluc protein. This suggested that the amount of CD81 in EVs was controlled, and EVs expressing CD81-Nluc were expected to be taken up similarly to normal EVs. Note that the Nluc activity shown in Figure 4B and 4C does not indicate simply the degree of uptake, but rather the combination of uptake and degradation/recycling of EVs. Time course analysis revealed that Nluc activity in both ACs and MG peaked at 4 h and was stable after 8 h (Figure 4B). These results suggest that uptake and decomposition after 8 h were in equilibrium. Because we found stable Nluc activity after 8 h, the uptake of EVs derived from LUHMES cells was compared between ACs and MG at 8 h. As shown in Figure 4C, EV uptake by ACs was higher than that by MG. This suggests that ACs receive more information from LUHMES cells via EVs than MG do.

### 3.4. Effect of IL-6 on EV Secretion and Uptake

**Figure 5:**
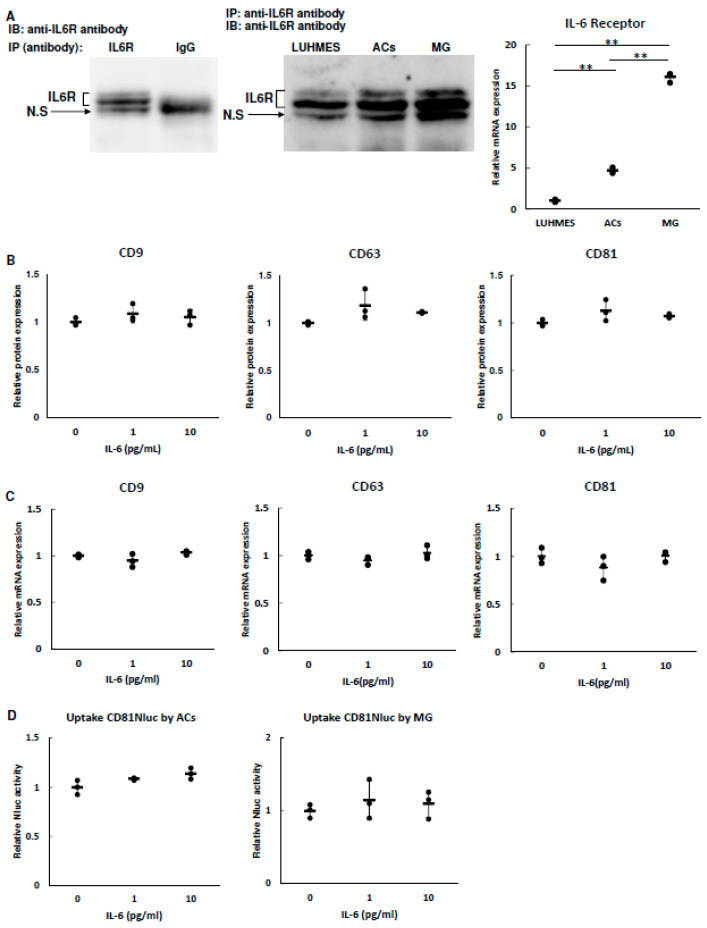
Effects of IL-6 on tetraspanin expression and uptake of EVs. (**A**) IL-6R expression in each cell was analyzed using Western blotting and qRT-PCR. Each cell lysate was immunoprecipitated with anti-IL-6R or anti-IgG antibodies and immunoblotted with anti-IL-6R ((**left**,**middle**) panels). Expression levels of IL-6R in each cell were analyzed using qRT-PCR ((**right**) panel). Data were obtained from three independent experiments (*n* = 3), ** *p* < 0.01. Error bars represent standard deviation. (**B**) A total of 2 × 10^5^ cells were seeded into a 12-well plate. The next day, the cells were washed with PBS and supplemented with 1 mL of F20 with 1 or 10 pg/mL IL-6. After 24 h, culture medium was collected and passed through a 220 nm filter. Each tetraspanin in the medium was measured using ELISA and each value was normalized using the cellular ATP activity as an internal standard. Data were obtained from three independent experiments (*n* = 3). Error bars represent standard deviation. (**C**) The expression levels of each tetraspanin in LUHMES cells treated with 1 or 10 pg/mL IL-6 were analyzed by qRT-PCR. Data were obtained from three independent experiments (*n* = 3). Error bars represent standard deviation. (**D**) A total of 2 × 10^5^ cells were seeded into a 12-well plate and cultured with medium containing LUHMES-derived CD81-Nluc expressed in EVs with 1 or 10 pg/mL IL-6 for 8 h. The background was subtracted from each value of Nluc activity and the result was normalized using cellular ATP activity as an internal standard. Data were obtained from three independent experiments (*n* = 3). Error bars represent standard deviation.

IL-6 is well known to be induced by stress. We thus first evaluated the expression level of IL-6R in LUHMES cells, ACs, and MG. Since no antibody specific to the IL-6R protein was available, immunoprecipitation was performed with an anti-IL-6 antibody and Western blotting was performed with the same antibody. As shown in Figure 5A, we observed three signals and found that the middle major signal and upper weak signal were derived from IL-6R and a lower signal was nonspecific. All three cell types expressed the IL-6R protein. To compare IL-6R expression among the cell types, mRNA was quantified by qRT-PCR. IL-6R mRNA expression was highest in MG and lowest in LUHMES cells. Next, we analyzed whether IL-6 affected the secretion of LUHMES-cell-derived EVs and their uptake into ACs and MG. The concentration of IL-6 selected for administration in this experiment was based on a meta-analysis [12]. We considered that 0 pg/mL was not a physiological state because healthy people also have about 1 pg/mL IL-6 (up to about 5 pg/mL). Meanwhile, IL-6 tends to be higher in depressed individuals than in healthy subjects, but it was expected that it would rarely exceed 10 pg/mL. Based on these results, 1 pg/mL IL-6 was set for the control group. As shown in Figure 5B,C, the expression of tetraspanin proteins in EVs from LUHMES cells and the cellular expression of tetraspanin mRNA in LUHMES cells were not affected by IL-6. Additionally, analysis of EV uptake showed no changes of Nluc activity in ACs and MG (Figure 5D). These results suggest that IL-6 is not involved in the degree of EV secretion and uptake.

### 3.5. IL-6 Affects the Amounts of miRNAs in EVs

Because EV secretion and uptake were not affected by IL-6, we focused on the expression ratio of each miRNA in the EVs. LUHMES cells were treated with 1 or 10 pg/mL IL-6 and then EVs in the culture supernatant were fractioned using nanofiltration. Total RNAs were eluted from the filters, and microarray analysis was performed. We found that many miRNAs were increased or decreased. To identify significant miRNAs, we selected them using the following criteria: (1) expression in LUHMES-derived EVs was >100 when 10 pg/mL IL-6 was applied; (2) expression of IL-6 at 10 pg/mL was more than twice that at 1 pg/mL; (3) expression in ACs and MG was <100. Four miRNAs were identified, and three miRNAs (hsa-miR-135a-3p, hsa-miR-6790-3p, and hsa-miR-11399) in EVs treated with 10 pg/mL IL-6 were significantly increased compared with those in EVs treated with 1 pg/mL (Table 1). Interestingly, these miRNAs were not very high in ACs or MG.

### 3.6. Target Analysis of Hsa-miR

Hsa-miRs in EVs derived from LUHMES cells were increased by IL-6. This result suggests that these miRNAs were increased in ACs and MG after uptake of EVs. Next, we introduced these miRNAs into ACs and MG via lipofection, and then microarray analysis of cellular mRNA was performed to identify target genes. The conditions for selection were as follows: (1) mRNA expression in the control group (introduction of ctrl miRNA) of >100; (2) mRNA expression at the time of introduction of each miRNA/mRNA expression at the time of introduction of ctrl miRNA of <0.5. The number of mRNAs that met the above criteria is shown in Table 2. mRNAs with expressions that were <0.5 for the same miRNAs in both of the two cell types are shown in Table 3. As shown in Table 2 and Table 3, the number of mRNAs decreased by hsa-miR-135-3p was the lowest. Additionally, mRNAs with expressions that were <0.5 for different miRNAs in the same cell type are shown in Table 4. Four mRNAs, *NREP* and *RPEL1* in ACs, and *CTNND1* and *KCTD12* in MG, were commonly suppressed by two miRNAs. mRNAs that were simultaneously suppressed by three miRNAs were not observed. We identified mRNAs involved in the nervous system using the data in Table 3 and Table 4, and searched for the target sequence of each miRNA. The database from which the nucleotide sequences and target sites were retrieved is shown in Table 5.

## 4. Discussion

There are various methods to collect EVs, but none have been optimized. EVs were first collected using ultracentrifugation and subsequently using gel filtration and capture methods using antibodies [24]. In this study, EVs were collected via a microfiltration method using 220 and 20 nm syringe filters (termed nanofiltration). The advantage of this method is that particles of uniform size are collected, but the sample is contaminated by microvesicles of 100–220 nm in size. Additionally, there are concerns that filter pores may be blocked by EVs, and that EVs may be destroyed by the membrane pressure [25,26]. Therefore, we examined cell destruction after passing through the filter, and confirmed that cells were not destroyed at a rate of two drops or less per second. Because EVs are smaller than cells, they are less likely to be destroyed when they pass through a syringe filter. We also believe that only approximately 5 min to pass 10 mL of medium through a 13 mm syringe filter of 50 nm in size is very useful for analysis. EVs trapped in a syringe filter can be easily eluted using phenol or detergent, but intact EVs cannot be recovered.

IL-6 is a proinflammatory cytokine that is increased by stress and involved in the development of depression [4]. Because there are reports that dysregulation of the dopamine system is involved in the pathophysiology of depression [27,28,29], in this study we investigated how IL-6 affects dopaminergic neuronal cells (LUHMES), astrocytes, and microglia. In particular, we applied IL-6 to neural precursor cells and analyzed quantitative and qualitative changes in EVs released by these cells as well as uptake of the EVs by astrocytes and microglia. First, we analyzed the intracellular proteins CD9, CD63, and CD81 and their mRNAs in EVs under normal culture conditions. The proteins and mRNAs of these tetraspanins were not correlated. It was suggested that the expression of each tetraspanin and its localization to EVs differs depending on the cell.

Tetraspain fused with a sensitive luciferase such as Nluc is used for in vitro and in vivo assays [30]. In this study, nanoluciferase-fused CD81 was expressed in EVs and analyzed for uptake by other cells. Intracellular uptake of EVs released by neural precursor cells peaked at 4 h and reached an equilibrium after 8 h in all cell types. Additionally, astrocytes had the highest uptake, and microglia showed a similar uptake to neural precursor cells. In vivo analysis has shown that retinal ganglion cells and microglia exhibit maximum fluorescence at 14 and 7 days, respectively, after administration of fluorescently labeled EVs in rats [31]. Therefore, several days may be required for EVs to reach cells in animals. Our results suggest that EVs released by neighboring cells are taken up within hours. It is thought that the difference in the uptake degree depending on the cell type is influenced by the combination of adhesion molecules expressed on the EV membrane and the plasma membrane by each cell type. For example, Horibe et al. reported that EV endocytosis depends on the expression level of related adhesion molecules [32]. This report suggests that the mechanism of EV uptake via endocytosis depends on the recipient cell type, which is consistent with the difference in EV uptake between astrocytes and microglia.

In this study, we found that IL-6 does not affect EV secretion or uptake. TNFα and IL-1β modify the miRNA cargo of astrocyte-shed EVs [33] and IL-6 stimulation results in high production of miR-455-3p by human umbilical cord mesenchymal stem cells [34]. Therefore, we focused on miRNAs among the molecules contained in EVs and comprehensively analyzed neural-precursor-cell-derived exosomal miRNAs of which the expression was increased or decreased by IL-6. Although many miRNAs were increased or decreased, we identified three miRNAs (hsa-miR-135a-3p, hsa-miR-6790-3p, and hsa-miR-11399) that were significantly increased dose-dependently by IL-6. Unfortunately, we did not find any studies of these miRNAs in relation to depression or psychological stress. Interestingly, there is a report that hsa-miR-135a-5p is weakly expressed in depressed patients [35] and that depressed patients with high expression levels of miR-135a-5p respond better to antidepressant treatment and are more likely to remain in remission [36].

We found that the expression levels of many genes were increased or decreased by transfection with each miRNA. Among them, *NREP* mRNA was decreased by both hsa-miR-6790-3p and hsa-miR-11399. In the Target Scan ver. 8.0 database, *NREP* is directly targeted by hsa-miR-6790-3p, but not hsa-miR-11399. Using Emboss water to match locations from each sequence (https://www.ebi.ac.uk/Tools/psa/emboss_water/) accessed on 16 August 2022, revealed that hsa-miR-11399 might directly target NREP with a high identity of 75%. These results suggest that the *NREP* gene might be targeted by both hsa-miR-6790-3p and hsa-miR-11399. *NREP*, which is also known as P311, promotes axonal regeneration [37] and cellular differentiation [38], and ectopic expression of *NREP* augments the motility of glioma cells [39]. These findings suggest that IL-6-related miRNAs in EVs may suppress the neuronal function of ACs.

In the present study, three miRNAs were transfected into MG and *KCTD12* was found to be reduced by both hsa-miR-6790-3p and hsa-miR-11399. *KCTD12* is a direct target of hsa-miR-6790-3p (Target Scan ver. 8.0). However, hsa-miR-11399 is not in the Target Scan database. Searching Emboss water revealed 48% homology between *KCTD12* and hsa-miR-11399. *KCTD12* is an important auxiliary subunit in GABAB receptor signaling [40]. *KCTD12* expression in peripheral blood mononuclear cells decreases under chronic stress [41]. Conversely, *KCTD12* expression in the circulating CD11b+ macrophages of bipolar disorder patients is significantly higher as a percentage than that in healthy controls [42]. Furthermore, a well-validated mouse model of chronic social duet stress revealed an increase of *KCTD12* expression in the dentate gyrus, a subregion of the hippocampus [43]. Thus, no consensus has been reached regarding *KCTD12* expression during stress. Although KCTD expression is widely spread over many areas of the brain, its distribution has been reported to vary by brain region and intracellular location. We think that this may explain why KCTD expression exhibits diverse responses to stress. [44,45].

We identified several mRNAs that were commonly decreased in ACs and MG by each miRNA (Table 3). Among them, *LLPH* is a direct target of hsa-miR-6790-3p (Target Scan ver. 8.0). *LLPH* has been predicted to be involved in dendrite extension and positive regulation of dendritic spine development. In mice, LLP is strongly expressed in the developing nervous system and its application to cultured neurons promotes dendrite outgrowth [46]. It is possible that the suppression of *LLPH* inhibits neuronal dendrite outgrowth in situations of elevated IL-6, i.e., chronic neuroinflammation, as was the condition in this study. This supports the idea that an altered neurostructure is part of the etiology of depression [4].

*CTNND1* was commonly downregulated in ACs and MG by hsa-miR-11399 and by hsa-miR-6790-3p in MG. In Target Scan, *CTNND1* is listed for hsa-miR-6790-3p and hsa-miR-11399, but in the Emboss water analysis, hsa-miR-6790-3p and hsa-miR-11399 exhibited 53.8% and 65.0% identity with *CTNND1*, respectively. *CTNND1* encodes p120-catenin. p120-catenin promotes reorganization of the actin cytoskeleton during the formation of cell adhesions [47]. Interestingly, wild-type astrocytes have a mesenchymal phenotype (i.e., E-cadherin negative and highly motile) and *CTNND1* phosphorylation regulates their mesenchymal–epithelial transition [48]. These data suggest that decreased expression of *CTNND1* impairs the MET function.

## 5. Conclusions

This study utilized nanofiltration to capture EVs in a culture medium. Nanofiltration has the advantage of easily capturing EVs, making it useful for analyzing proteins and nucleic acids in EVs. However, since the EVs captured by the filter cannot be collected in an intact condition, it is difficult to analyze their in vitro or in vivo functions. Our findings suggest that the uptake of IL-6-derived EVs secreted from neural precursor cells by ACs and MG may suppress the expression of genes involved in neural regeneration, indicating that high levels of IL-6, associated with depression, contribute to decreased neuronal functions via miRNAs in EVs.

## Figures and Tables

**Table 1:** Expression of miRNAs in EVs and in cells.

miRNA IDs		miRNA Expression in EVs			miRNA Expression in Cells	
	1 pg/mL IL-6	10 pg/mL IL-6	Ratio *	*p*-Value **	ACs	MG
hsa-miR-135a-3p	120.4 (±25.1)	284.5 (±39.5)	2.4	0.0495	17.4 (±6.4)	48.0 (±5.2)
hsa-miR-11399	242.3 (±81.4)	538.8 (±151.4)	2.2	0.0495	15.9 (±5.5)	32.6 (±3.1)
hsa-miR-6790-3p	176.4 (±89.3)	383.5 (±29.0)	2.2	0.0495	26.5 (±9.3)	67.3 (±4.6)

* miRNA expression of 10 pg/mL IL-6/miRNA expression of 1 pg/mL IL-6. Mean (± standard deviation), *n* = 3. ** Wilcoxon signed-rank test.

**Table 2:** Number of mRNAs for which expression was less than 1/2 due to transfection of miRNAs.

Cell	Transfected miRNA	Number of mRNAs
ACs	hsa-miR-135-3p	2
	hsa-miR-6790-3p	15
	hsa-miR-11399	547
MG	hsa-miR-135-3p	13
	hsa-miR-6790-3p	34
	hsa-miR-11399	205
Common	hsa-miR-135-3p	1
	hsa-miR-6790-3p	10
	hsa-miR-11399	47

**Table 3:** mRNAs for which expression was less than 1/2 in both ACs and MG due to transfection of miRNAs.

Transfected miRNAs	mRNAs Expression Less than 1/2		
hsa-miR-135-3p	UBE2D3		
hsa-miR-6790-3p	*MINOS1-NBL1*	*MRPL11*	
	*PTS*	*RPEL1*	
	*RAB6A*	*LLPH*	
	*RPE*	*IMP4*	
	*SET*	*EI24*	
hsa-miR-11399	*ACTR3*	*TOR1AIP1*	*BCCIP*
	*CDKN1A*	*AMD1*	*SERINC1*
	*CENPF*	*GATC*	*SOAT1*
	*NCKAP1*	*ANXA1*	*TOP2A*
	*SUPT16H*	*IPO5*	*HIST2H2AA4*
	*MRFAP1L1*	*LIMS1*	*CHCHD7*
	*CREB1*	*FAM45A*	*HIST1H2BG*
	*CTNND1*	*ZNF117*	*HIST1H2BH*
	*ARL6IP6*	*LARS*	*CASP3*
	*ZNF836*	*ZFR*	*MARVELD1*
	*ZNF721*	*POLR2B*	*WDR75DGKZ*
	*EFNB2*	*ANLN*	*USO1PRPF4B*
	*RHOBTB3*	*ZFAND6*	*HIST3H2A*
	*PUM2*	*NDFIP2*	*TTC37*
	*SF3B1*	*NOL8*	*G3BP2*
	*BAMBI*	*EEF1A1P31*	

**Table 4:** Common mRNAs reduced by less than 1/2 following transfection of different miRNAs in cells of the same species.

Gene	hsa-miR-135-3p	hsa-miR-6790-3p	hsa-miR-11399-3p
*NREP* of ACs	NE	↓	↓
*RPEL1* of ACs	NE	↓	↓
*CTNND1* of MG	NE	↓	↓
*KCTD12* of MG	NE	↓	↓↓

↓: less than 1/2, ↓↓: less than 1/10, NE: no effect.

**Table 5:** Comparison of alignment using Target Scan or Emboss water.

miRNA Name	hsa-miR-135a-3p	hsa-miR-11399	hsa-miR-6790-3p
miRNA sequence	UAUAGGGAUUGGAGCCGUGGCG	UUCAGGUCUGGGGCUGAAACCU	CGACCUCGGCGACCCCUCACU
*NREP* (Target Scan)	none	none	UGUAUGGGUAUUGAUGAGGUCAU
*NREP* (Emboss water)	none	GAUGCUGCCACAGGACCUGA	GUGUAUGGGUAUUGAUGAGGUC
*KCTD12* (Target Scan)	none	none	CUCCAAGCGCCGUAGGAGGUCAC
*KCTD12* (Emboss water)	none	GGUUGCAGCUCCUGAGUGCAGCGCGGCUUCCUG	GCCCGGGGCCGCCGCCCUCG
*LLPH* (Target Scan)	none	none	GAGGCAGGUGGAUCACGAGGUCA
*LLPH* (Emboss water)	none	none	UGAGGCAGAUGGAUCACCUGAGGUC
*CTNND1* (Target Scan)	none	none	none
*CTNND1* (Emboss water)	none	GGCUCCGCCCCUUACCUUCA	AGAGAGGAGUGAAGCUCGCCGGAAAC

Underlined bases indicate miRNA target alignment. Below the mRNA is the name of the database from which the miRNA target was retrieved.

## Data Availability

Not applicable.

## References

[1] Wang J., Wu X., Lai W., Long E., Zhang X., Li W., Zhu Y., Chen C., Zhong X., Liu Z. (2017). Prevalence of Depression and Depressive Symptoms among Outpatients: A Systematic Review and Meta-Analysis. BMJ Open.

[2] Guilbert J.J. (2003). The World Health Report 2002—Reducing Risks, Promoting Healthy Life. Educ. Health Abingdon Engl..

[3] McCarron R.M., Shapiro B., Rawles J., Luo J. (2021). Depression. Ann. Intern. Med..

[4] Beurel E., Toups M., Nemeroff C.B. (2020). The Bidirectional Relationship of Depression and Inflammation: Double Trouble. Neuron.

[5] Ongür D., Drevets W.C., Price J.L. (1998). Glial Reduction in the Subgenual Prefrontal Cortex in Mood Disorders. Proc. Natl. Acad. Sci. USA.

[6] Eroglu C., Barres B.A. (2010). Regulation of Synaptic Connectivity by Glia. Nature.

[7] Nagy C., Suderman M., Yang J., Szyf M., Mechawar N., Ernst C., Turecki G. (2015). Astrocytic Abnormalities and Global DNA Methylation Patterns in Depression and Suicide. Mol. Psychiatry.

[8] Nie X., Kitaoka S., Tanaka K., Segi-Nishida E., Imoto Y., Ogawa A., Nakano F., Tomohiro A., Nakayama K., Taniguchi M. (2018). The Innate Immune Receptors TLR2/4 Mediate Repeated Social Defeat Stress-Induced Social Avoidance through Prefrontal Microglial Activation. Neuron.

[9] Rose-John S. (2018). Interleukin-6 Family Cytokines. Cold Spring Harb. Perspect. Biol..

[10] Qing H., Desrouleaux R., Israni-Winger K., Mineur Y.S., Fogelman N., Zhang C., Rashed S., Palm N.W., Sinha R., Picciotto M.R. (2020). Origin and Function of Stress-Induced IL-6 in Murine Models. Cell.

[11] Steptoe A., Hamer M., Chida Y. (2007). The Effects of Acute Psychological Stress on Circulating Inflammatory Factors in Humans: A Review and Meta-Analysis. Brain. Behav. Immun..

[12] Dowlati Y., Herrmann N., Swardfager W., Liu H., Sham L., Reim E.K., Lanctôt K.L. (2010). A Meta-Analysis of Cytokines in Major Depression. Biol. Psychiatry.

[13] Brietzke E., Scheinberg M., Lafer B. (2011). Therapeutic Potential of Interleukin-6 Antagonism in Bipolar Disorder. Med. Hypotheses.

[14] Mohr A.M., Mott J.L. (2015). Overview of MicroRNA Biology. Semin. Liver Dis..

[15] Costa Verdera H., Gitz-Francois J.J., Schiffelers R.M., Vader P. (2017). Cellular Uptake of Extracellular Vesicles Is Mediated by Clathrin-Independent Endocytosis and Macropinocytosis. J. Control. Release.

[16] He C., Zheng S., Luo Y., Wang B. (2018). Exosome Theranostics: Biology and Translational Medicine. Theranostics.

[17] Valadi H., Ekström K., Bossios A., Sjöstrand M., Lee J.J., Lötvall J.O. (2007). Exosome-Mediated Transfer of MRNAs and MicroRNAs Is a Novel Mechanism of Genetic Exchange between Cells. Nat. Cell Biol..

[18] Schiller E.A., Cohen K., Lin X., El-Khawam R., Hanna N. (2023). Extracellular Vesicle-MicroRNAs as Diagnostic Biomarkers in Preterm Neonates. Int. J. Mol. Sci..

[19] Rahimian N., Nahand J.S., Hamblin M.R., Mirzaei H. (2023). Exosomal MicroRNA Profiling. Methods Mol. Biol. Clifton NJ.

[20] Upadhya R., Zingg W., Shetty S., Shetty A.K. (2020). Astrocyte-Derived Extracellular Vesicles: Neuroreparative Properties and Role in the Pathogenesis of Neurodegenerative Disorders. J. Control. Release Off. J. Control. Release Soc..

[21] Jotatsu T., Izumi H., Morimoto Y., Yatera K. (2020). Selection of MicroRNAs in Extracellular Vesicles for Diagnosis of Malignant Pleural Mesothelioma by in Vitro Analysis. Oncol. Rep..

[22] Yamaguchi T., Kurita T., Nishio K., Tsukada J., Hachisuga T., Morimoto Y., Iwai Y., Izumi H. (2015). Expression of BAF57 in Ovarian Cancer Cells and Drug Sensitivity. Cancer Sci..

[23] Murakami M., Izumi H., Kurita T., Koi C., Morimoto Y., Yoshino K. (2020). UBE2L6 Is Involved in Cisplatin Resistance by Regulating the Transcription of ABCB6. Anti-Cancer Agents Med. Chem..

[24] Sidhom K., Obi P.O., Saleem A. (2020). A Review of Exosomal Isolation Methods: Is Size Exclusion Chromatography the Best Option?. Int. J. Mol. Sci..

[25] Zhu L., Sun H.-T., Wang S., Huang S.-L., Zheng Y., Wang C.-Q., Hu B.-Y., Qin W., Zou T.-T., Fu Y. (2020). Isolation and Characterization of Exosomes for Cancer Research. J. Hematol. Oncol..

[26] Doyle L.M., Wang M.Z. (2019). Overview of Extracellular Vesicles, Their Origin, Composition, Purpose, and Methods for Exosome Isolation and Analysis. Cells.

[27] Baik J.-H. (2020). Stress and the Dopaminergic Reward System. Exp. Mol. Med..

[28] Takamura N., Nakagawa S., Masuda T., Boku S., Kato A., Song N., An Y., Kitaichi Y., Inoue T., Koyama T. (2014). The Effect of Dopamine on Adult Hippocampal Neurogenesis. Prog. Neuropsychopharmacol. Biol. Psychiatry.

[29] Suneson K., Lindahl J., Chamli Hårsmar S., Söderberg G., Lindqvist D. (2021). Inflammatory Depression-Mechanisms and Non-Pharmacological Interventions. Int. J. Mol. Sci..

[30] Gupta D., Liang X., Pavlova S., Wiklander O.P., Corso G., Zhao Y., Saher O., Bost J., Zickler A.M., Piffko A. (2020). Quantification of Extracellular Vesicles in Vitro and in Vivo Using Sensitive Bioluminescence Imaging. J. Extracell. Vesicles.

[31] Mathew B., Torres L.A., Gamboa Acha L., Tran S., Liu A., Patel R., Chennakesavalu M., Aneesh A., Huang C.-C., Feinstein D.L. (2021). Uptake and Distribution of Administered Bone Marrow Mesenchymal Stem Cell Extracellular Vesicles in Retina. Cells.

[32] Horibe S., Tanahashi T., Kawauchi S., Murakami Y., Rikitake Y. (2018). Mechanism of Recipient Cell-Dependent Differences in Exosome Uptake. BMC Cancer.

[33] Chaudhuri A.D., Dastgheyb R.M., Yoo S.-W., Trout A., Talbot C.C., Hao H., Witwer K.W., Haughey N.J. (2018). TNFα and IL-1β Modify the MiRNA Cargo of Astrocyte Shed Extracellular Vesicles to Regulate Neurotrophic Signaling in Neurons. Cell Death Dis..

[34] Shao M., Xu Q., Wu Z., Chen Y., Shu Y., Cao X., Chen M., Zhang B., Zhou Y., Yao R. (2020). Exosomes Derived from Human Umbilical Cord Mesenchymal Stem Cells Ameliorate IL-6-Induced Acute Liver Injury through MiR-455-3p. Stem Cell Res. Ther..

[35] Issler O., Haramati S., Paul E.D., Maeno H., Navon I., Zwang R., Gil S., Mayberg H.S., Dunlop B.W., Menke A. (2014). MicroRNA 135 Is Essential for Chronic Stress Resiliency, Antidepressant Efficacy, and Intact Serotonergic Activity. Neuron.

[36] Marshe V.S., Islam F., Maciukiewicz M., Fiori L.M., Yerko V., Yang J., Turecki G., Foster J.A., Kennedy S.H., Blumberger D.M. (2020). Validation Study of MicroRNAs Previously Associated with Antidepressant Response in Older Adults Treated for Late-Life Depression with Venlafaxine. Prog. Neuropsychopharmacol. Biol. Psychiatry.

[37] Fujitani M., Yamagishi S., Che Y.H., Hata K., Kubo T., Ino H., Tohyama M., Yamashita T. (2004). P311 Accelerates Nerve Regeneration of the Axotomized Facial Nerve. J. Neurochem..

[38] Pan D., Zhe X., Jakkaraju S., Taylor G.A., Schuger L. (2002). P311 Induces a TGF-Beta1-Independent, Nonfibrogenic Myofibroblast Phenotype. J. Clin. Investig..

[39] Mariani L., McDonough W.S., Hoelzinger D.B., Beaudry C., Kaczmarek E., Coons S.W., Giese A., Moghaddam M., Seiler R.W., Berens M.E. (2001). Identification and Validation of P311 as a Glioblastoma Invasion Gene Using Laser Capture Microdissection. Cancer Res..

[40] Seddik R., Jungblut S.P., Silander O.K., Rajalu M., Fritzius T., Besseyrias V., Jacquier V., Fakler B., Gassmann M., Bettler B. (2012). Opposite Effects of KCTD Subunit Domains on GABA(B) Receptor-Mediated Desensitization. J. Biol. Chem..

[41] Miller G.E., Chen E., Sze J., Marin T., Arevalo J.M.G., Doll R., Ma R., Cole S.W. (2008). A Functional Genomic Fingerprint of Chronic Stress in Humans: Blunted Glucocorticoid and Increased NF-KappaB Signaling. Biol. Psychiatry.

[42] Wu T.-N., Chen C.-K., Lee C.-S., Wu B.-J., Sun H.-J., Chang C.-H., Chen C.-Y., Wu L.S.-H., Cheng A.T.-A. (2019). Lithium and GADL1 Regulate Glycogen Synthase Kinase-3 Activity to Modulate KCTD12 Expression. Sci. Rep..

[43] Deng S.-L., Hu Z.-L., Mao L., Gao B., Yang Q., Wang F., Chen J.-G. (2021). The Effects of Kctd12, an Auxiliary Subunit of GABAB Receptor in Dentate Gyrus on Behavioral Response to Chronic Social Defeat Stress in Mice. Pharmacol. Res..

[44] Schwenk J., Metz M., Zolles G., Turecek R., Fritzius T., Bildl W., Tarusawa E., Kulik A., Unger A., Ivankova K. (2010). Native GABA(B) Receptors Are Heteromultimers with a Family of Auxiliary Subunits. Nature.

[45] Metz M., Gassmann M., Fakler B., Schaeren-Wiemers N., Bettler B. (2011). Distribution of the Auxiliary GABAB Receptor Subunits KCTD8, 12, 12b, and 16 in the Mouse Brain. J. Comp. Neurol..

[46] Yu N.-K., Kim H.F., Shim J., Kim S., Kim D.W., Kwak C., Sim S.-E., Choi J.-H., Ahn S., Yoo J. (2016). A Transducible Nuclear/Nucleolar Protein, MLLP, Regulates Neuronal Morphogenesis and Synaptic Transmission. Sci. Rep..

[47] Castaño J., Solanas G., Casagolda D., Raurell I., Villagrasa P., Bustelo X.R., García de Herreros A., Duñach M. (2007). Specific Phosphorylation of P120-Catenin Regulatory Domain Differently Modulates Its Binding to RhoA. Mol. Cell. Biol..

[48] Yang J., Bassuk A.G., Merl-Pham J., Hsu C.-W., Colgan D.F., Li X., Au K.S., Zhang L., Smemo S., Justus S. (2016). Catenin Delta-1 (CTNND1) Phosphorylation Controls the Mesenchymal to Epithelial Transition in Astrocytic Tumors. Hum. Mol. Genet..

